# The mystery of the COVID toes – turning evidence-based medicine on its head

**DOI:** 10.1186/s13047-020-00408-w

**Published:** 2020-06-23

**Authors:** Ivan R. Bristow, Alan M. Borthwick

**Affiliations:** 1 Visiting Fellow, School of Health Sciences, Faculty of Environmental and Life Sciences, University of Southampton, Highfield, Southampton, SO17 1BJ UK; 2 Emeritus Professor, School of Health Sciences, Faculty of Environmental and Life Sciences, University of Southampton, Highfield, Southampton, SO17 1BJ UK

**Keywords:** COVID-19, Evidence based medicine, Case study

## Abstract

The recent and rapid emergence of COVID-19 infection has led to a flood of publications describing all aspects of the disease and its presentation. The appearance of chilblain-like lesions, in children and young adults has particularly caught the attention of healthcare professionals with an interest in the foot. With such a novel infection, demand for information is high at a time when evidence is scarce. Consequently, there has been a renaissance in the publication of case studies. This type of research, previously relegated from many mainstream journals, as a low level source of evidence, has permitted the rapid reporting, publication and dissemination of much needed clinical data which can be used as a foundation to inform further research and investigations about a new global infection.

## Background

It has been 2 months since reports began to appear in the medical literature and on social media discussing the appearance of chilblain like lesions on the toes of children and young adults [1, 2]. The phenomenon received a lot of coverage in the media - at a time when available evidence was in short supply. The emergence of the COVID-19 pandemic has presented a new challenge. For many years, healthcare professionals have been trained in the use of evidence-based medicine to understand and guide their decisions and management of patient pathologies. Judgement on the quality of the evidence is made through assessment of the methodology of the work and ranking it within the hierarchy of evidence [3]. High level evidence from clinical trials and systematic reviews are held as the gold standard for informing clinicians, but what happens when they do not exist? With COVID-19, clinicians are faced with a disease where little is known but the need for knowledge is high. In the light of this, we have seen the re-emergence of a type of paper which had previously been threatened with extinction in mainstream medical journals [4] – the humble case report and case series.

## Main text

Case reports were a common publication in many medical and healthcare journals in previous decades but their existence in the literature was increasingly questioned over their utility in modern healthcare [5] as evidence based medicine emerged in the late 1980’s. With the establishment of the hierarchy of evidence they were firmly placed at the bottom [6]. It soon followed that many medical journals sought to reduce or entirely remove them. They were not so frequently cited as other publication types [7] and so could potentially reduce a journal’s impact factor. Subsequently, many publishers and professional medical organisations created open access, online platforms accepting case studies separate from their main journals.

With the global emergence of COVID-19, the world was exposed to a new type of infection, about which very little was known in terms of its presentation, clinical features and management. With the levels of mortality occurring with the infection, the need for clinical data was urgent. Consequently, journals, with the lack of any high-level evidence, had to turn to expert commentary and begin to piece together the COVID-19 puzzle together using case reports and case series. In fact, up to June 5th, a search of PUBMED using the operator terms “Case [title]” AND “corona OR covid” yields over 700 papers published this year.

In April, early papers reported the emergence of a suspected phenomenon of chilblain-like lesions appearing in children and young adults [1, 2]. It was reported as ‘suspected’, as the subjects were not able to be fully assessed or indeed even confirmed as having had the infection. With increasing interest in these lesions, national newspapers in many countries were reporting these as potential signs of the disease of children and further medical publications rapidly followed [8–14] on a daily basis. All of these were case reports and case series. To create a clearer picture of how the virus affected the skin, additional patient data was requested from clinicians globally and collected by the American Academy of Dermatology and the British Association of Dermatologists to try and collate a clearer clinical picture of the phenomenon. Within a couple of months, the first dataset was published collating 318 cases of “COVID toe” [15].

The survey collected data primarily from physicians, with contributions from other healthcare professionals, consisted of collated case reports. Its conclusion highlighted the limitations of the case study methodology. From the data the prevalence was not able to be estimated, causation was not able to be proven (many subjects had not undergone any testing for the infection) and only hypotheses could be generated as to the natural history of the disease and its pathophysiology. Retrospective bias was also an issue. However, despite this, at this time the hidden power of case study in rapidly reporting new phenomenon, diseases and clinical observations [16] has been used to its fullest extent from which further research and investigation can be based as the foundation of new knowledge.

## Conclusion

Consequently, the emergence of a global epidemic has served to highlight how all types and levels of evidence can have utility and relevance in modern healthcare. Even with known limitations, they can fulfil an urgent need to progress knowledge in times of need.

## Data Availability

Not applicable.
